# HIV infection and sexual risk behaviour among youth who have experienced orphanhood: systematic review and meta-analysis

**DOI:** 10.1186/1758-2652-14-25

**Published:** 2011-05-18

**Authors:** Don Operario, Kristen Underhill, Carolyn Chuong, Lucie Cluver

**Affiliations:** 1 Department of Community Health, Brown University, Providence, RI, USA; 2 Department of Social Policy and Social Work, Oxford University, Oxford, UK

## Abstract

**Background:**

Previous research has suggested that orphaned children and adolescents might have elevated risk for HIV infection. We examined the state of evidence regarding the association between orphan status and HIV risk in studies of youth aged 24 years and younger.

**Methods:**

Using systematic review methodology, we identified 10 studies reporting data from 12 countries comparing orphaned and non-orphaned youth on HIV-related risk indicators, including HIV serostatus, other sexually transmitted infections, pregnancy and sexual behaviours. We meta-analyzed data from six studies reporting prevalence data on the association between orphan status and HIV serostatus, and we qualitatively summarized data from all studies on behavioural risk factors for HIV among orphaned youth.

**Results:**

Meta-analysis of HIV testing data from 19,140 participants indicated significantly greater HIV seroprevalence among orphaned (10.8%) compared with non-orphaned youth (5.9%) (odds ratio = 1.97; 95% confidence interval = 1.41-2.75). Trends across studies showed evidence for greater sexual risk behaviour in orphaned youth.

**Conclusions:**

Studies on HIV risk in orphaned populations, which mostly include samples from sub-Saharan Africa, show nearly two-fold greater odds of HIV infection among orphaned youth and higher levels of sexual risk behaviour than among their non-orphaned peers. Interventions to reduce risk for HIV transmission in orphaned youth are needed to address the sequelae of parental illness and death that might contribute to sexual risk and HIV infection.

## Background

One of the many consequences of the global HIV epidemic is the impact of adult parental AIDS illness and death on children [1,2]. Orphans are defined as children under the age of 18 years whose mother, father or both parents have died [3]. By 2011, there will have been an approximately 142 million orphaned children worldwide, most of whom reside in the developing world, including sub-Saharan Africa and Asia [3]. Although there are important debates about defining and measuring orphanhood [4-6], international agencies have suggested that youth who have experienced orphanhood might have elevated risk for HIV infection through sexual transmission [3]. Indeed, because sexual debut generally occurs during adolescence or young adulthood, experiencing the death of a parent during this developmental period may contribute to riskier behaviours or a high-risk context for HIV infection [7,8].

Some of the challenges experienced by youth orphaned in the context of HIV/AIDS have been documented. Studies have observed associations between orphanhood status and poor educational outcomes [9-15]. Mental health problems among orphans are also apparent, including increased risk for depression, trauma and emotional distress [16-19]. Other studies report greater levels of poverty and economic disadvantage among orphaned children [20,21]. However, health and social vulnerabilities among orphaned youth have not been consistently documented across studies, and there have been noteworthy cautions against assuming that all orphaned youth face exceptionally greater risk than non-orphaned youth [5,6].

There have been claims that children of HIV-infected parents might be more likely to become infected with HIV through sexual risk behaviour [22,23]. Perinatal transmission is unlikely to explain the higher observed HIV prevalence among orphaned youth. The median survival age for perinatally infected infants is two years in the absence of antiretroviral treatment, which became available in many developing world settings only in the past decade [24]. Increased sexual risk behaviour is an alternative explanation for elevated HIV infection in youth who had experienced orphanhood. Indeed, educational shortfalls, mental health problems and poverty, which are associated with orphanhood, are also factors that are associated with sexual risk behaviour in youth populations [23,25,26].

We conducted a systematic review to examine the body of literature on HIV risk in youth aged 24 years and younger who have experienced the death of one or more parent. The goal of this review was to identify all published studies that have assessed HIV status or HIV-related risk behaviour in youth populations, and that compared HIV status and risk between participants who had or had not experienced orphanhood. Although we anticipated that the majority of studies would assess orphaned populations in high-HIV-prevalence countries, we searched for any studies that took place worldwide. We aimed to describe characteristics of identified studies, assess their methodological quality, and summarize findings on HIV-related risk across studies. We also aimed to conduct a meta-analysis of HIV prevalence in orphaned versus non-orphaned youth. We hypothesized that orphans would have a higher prevalence of HIV infection and self-reported sexual risk behaviour than non-orphans.

## Methods

### Study selection

We searched for any study assessing HIV serostatus or HIV-related behavioural risk factors among youth aged 24 years and younger, and which compared orphaned and non-orphaned subgroups within the study sample. Studies were included if they met all of the following criteria: (1) they assessed death of one or more parent; (2) they assessed at least one form of HIV risk (i.e., HIV infection, other sexually transmitted infection, pregnancy or sexual risk behaviour); and (3) they compared orphaned and non-orphaned participants on HIV-relevant variables. Study designs of interest were cross-sectional studies and longitudinal cohort studies; for longitudinal designs, baseline data were included. The investigators carried out all searches and procedures for study selection, data extraction and analysis.

### Search

Electronic searches of PubMed/Biomed Central/Medline, PsycINFO, and EMBASE were carried out initially in February 2009 and updated in June 2009, including studies from 1980 onwards. Our search strategy included MeSH terms for HIV and terms associated with orphan status, truncated where relevant [HIV* OR AIDS* OR HIV Infections[MeSH] OR HIV[MeSH] OR hiv[tw] OR hiv-1*[tw] OR hiv-2*[tw] OR hiv1[tw] OR hiv2[tw] OR hiv infect*[tw] OR human immunodeficiency virus[tw] OR human immunedeficiency virus[tw] OR human immuno-deficiency virus[tw] OR human immune-deficiency virus[tw] OR ((human immun*) AND (deficiency virus[tw])) OR acquired immunodeficiency syndrome[tw] OR acquired immunedeficiency syndrome[tw] OR acquired immuno-deficiency syndrome[tw] OR acquired immune-deficiency syndrome[tw] OR ((acquired immun*] AND (deficiency syndrome[tw])) OR "Sexually Transmitted Diseases, Viral"] AND [orphan* or OVC or vulnerable children or parental death or parental bereav*].

We did not use linguistic or geographical search restrictions, and we obtained English-language translations of articles where necessary. We cross-referenced previous reviews and primary studies for additional citations, and we contacted expert researchers to identify unpublished and forthcoming studies.

**Figure 1:**
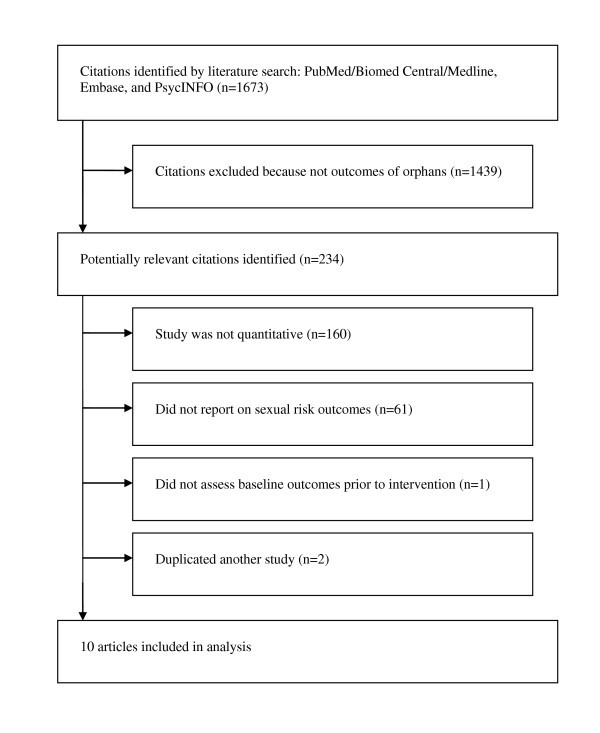
**Flowchart for systematic search**.

All identified records (n = 1673) were initially screened by one author to exclude citations that were clearly irrelevant. A short-list of records (n = 234) was prepared and reviewed independently by two authors. If either author found an article to be relevant, a full-text copy was obtained and assessed for inclusion. Studies were excluded because they did not report quantitative results (n = 160), did not report HIV or sexual risk variables (n = 61), did not report baseline risk variables prior to intervention (n = 1), or were duplicates of other studies (n = 2). Two independent assessors approved the final list of included studies (n = 10); disagreements about inclusion were resolved by discussion (see Figure 1 for flowchart of systematic review).

### Data extraction

Data were extracted by two trained, independent coders and included details about study design, sampling approach, participant characteristics, variables of interest, analysis and results (see Table 1). Coders showed 95% agreement or higher. For studies with multiple orphan subgroups (e.g., maternal, paternal or double orphans), all relevant data were abstracted. The authors were not blind to any aspect of the studies.

**Table 1:** Characteristics of included studies

Study	Location (year)	Study design	Sampling method	Sample characteristics	HIV-related variables
Birdthistle [29]	Harare (Highfield area), Zimbabwe (2004)	Cross-sectional	Representative household sampling	n = 863; females only; age range 14 to 20; participation rate = 67%	*Biological testing: *HIV status, HSV-2 *Self-report*: Pregnancy, ever had sex, >1 partner in lifetime, regular partner at time of interview, ever forced to have sex, ever had exchange sex, first sex was forced, first sexual partner 10+ years older, condom not used during first sex
Gregson [30]	Manicaland, Zimbabwe (2001-3)	Cross-sectional	Stratified population-based household sampling	n = 1523; males = 31%, females = 69%; age range for males 17 to 18; age range for females 15 to 18; participation rate = 75%	*Biological testing: *HIV infection *Self-report: *History of STI symptoms, pregnancy, ever had sex, currently married, more than one partner in lifetime
Kamali [38]	15 rural villages in Masaka district, Uganda (1989-1993)	Longitudinal	Sample included all consenting residents in the selected villages in 1989-90	n = 4975; included both males and females but percentages unclear; age range 0 to 15	*Biological testing: *HIV-1 infection testing carried out among 4594 participants
Kang [31]	Epworth and Chitungwiza (near Harare), Zimbabwe (year not known)	Cross-sectional	Convenience sampling	n = 196; females only; age range 16 to 19; participation rate = 98%	*Biological testing: *HIV infection, HSV-2 infection, pregnancy *Self-report: *History of STIs and pregnancy, ever had vaginal or anal sex, first sex was forced, had first sex because needed food/money/school fees, used contraceptive during first sex, current relationship is sexual, receives basic needs from partner, ever consumed alcohol, more than one partner in lifetime
Kissin [32]	St Petersburg, Russia (2006)	Cross-sectional	Systematic venue-based sampling	n = 313; males = 63%, females = 27%; age range 15 to 19; participation rate = 92%	*Biological testing: *HIV infection *Self-report: *Ever had sex, lifetime transactional sex, lifetime anal sex, past-year same-sex partner, past-year number of partners, lifetime STI diagnosis, pregnancy
McGrath [37]	Umkhanyakude district, KwaZulu-Natal, South Africa (2003-7)	Longitudinal	Representative household sampling	n = 8753; male = 46%, female = 54%; age range 12 to 25	*Self-report: *Ever had sex, age at first sex
Nyamukapa [33]	21 rural and urban districts in Zimbabwe (2004)	Cross-sectional	Purposive sampling of districts (on the basis of poverty and education); census enumeration areas selected according to size and geography; households within each enumeration area selected to fulfill quota	n = 4660; male = 51%, female = 49%; age range 12 to 17 years	*Self-report: *Ever had sex, early sexual intercourse, ever forced to have sex, ever engaged in high-risk sex
Operario [34]	All nine provinces in South Africa (2003)	Cross-sectional	National, representative household sampling	n = 11,904; male = 48%, female = 52%; age range 15 to 24; participation rate = 77%	*Biological testing: *HIV infection *Self-report: *STI in past year, pregnancy history, ever had oral sex, ever had vaginal sex, ever had anal sex, >1 sex partner in past year, last sex was unprotected, ever forced to have sex, ever had transactional sex
Palermo [35]	Benin (2006), Chad (2005), Congo (2005), Cote d'Ivoire (2005), Lesotho (2004), Malawi (2004), Mozambique (2003), Tanzania (2004), Uganda (2006), Zimbabwe (2005-6)	Cross-sectional	National, representative household sampling	Total n = 11,975 [range n = 711 (Cote d'Ivoire), n = 1801 (Benin)]; all females; age range 15 to 17	*Self-report: *Ever had sex, pregnancy
Thurman [36]	Durban Metro and Mtunzini district, KwaZulu-Natal, South Africa (2001)	Cross-sectional	Multi-stage cluster sampling approach; all households within selected census enumeration areas were contacted	n = 1694; male = 47%, female = 53%; age range 14 to 18 years; participation rate = 95%	*Self-report: *Ever had vaginal sex, relative age difference of current sex partner, more than one partner in past year, ever had transactional sex, condom used during last sex, had first sex at age 13 or under, first sexual partner age 17 or older, first sex was willing, first sex was persuaded, first sex was tricked, first sex was forced, condom used during first sex

### Analysis

We conducted a meta-analysis of HIV seroprevalence in orphaned versus non-orphaned participants using Review Manager version 5.0, a statistical software programme developed by the Nordic Cochrane Center for meta-analyzing data for systematic reviews [27]. We were unable to conduct meta-analysis on other HIV-related risk variables (history of sexually transmitted infections, pregnancy, sexual behaviours) due to between-study heterogeneity in variables; for these variables, trends across studies are described qualitatively.

We used the χ^2 ^test to assess between-study heterogeneity in HIV seroprevalence findings, and the I^2 ^statistic to assess the degree to which variability was due to between-study differences rather than chance. Effect sizes were estimated using odds risk (OR) ratios and 95% confidence intervals (CIs). ORs greater than 1.0 indicated an increased probability of HIV infection among orphaned compared with non-orphaned participants. There were insufficient data to meta-analyze data by type of orphan status (i.e., maternal orphans, paternal orphans and double orphans). We investigated publication bias using a visual inspection of funnel plots, and examined the stability of the meta-analysis results using Orwin's fail-safe N analysis.

### Assessment of methodological quality

We assessed methodological quality using components of the STROBE (Strengthening the Reporting of Observational Studies in Epidemiology) checklist, which outlines criteria for assessing studies using cross-sectional designs [28]. The following characteristics were appraised: (1) sampling approach; (2) assessment of independent variables; (3) comparability of independent variable subgroups; (4) assessment of dependent variables of interest; (5) participation rate; and (6) statistical analyses.

## Results

### Characteristics of included studies

This analysis includes 10 studies encompassing 46,856 participants recruited from 12 countries, mostly in sub-Saharan Africa (see Table 1). Included studies were published between 1996 and 2009. Eight studies reported cross-sectional surveys [29-36] and two studies reported longitudinal surveys [37,38]. One study, which reported sexual risk behaviour data on orphans, was excluded because it was a parenting intervention for people living with HIV that only included follow-up measures of children without reporting baseline data [39]. Sampling techniques included representative household sampling, systematic venue-based targeted sampling, and convenience sampling. Data were collected from Benin (number of studies [k] = 1), Chad (k = 1), Congo (k = 1), Cote d'Ivoire (k = 1), Lesotho (k = 1), Malawi (k = 1), Mozambique (k = 1), South Africa (k = 3), Russia (k = 1), Tanzania (k = 1), Uganda (k = 2), and Zimbabwe (k = 5).

One study conducted representative household surveys of female youth in 10 countries [35], reporting separate findings for each country. Sample sizes per unique survey ranged from 196 to 11,904; in the 10-country study, the aggregate sample size was 11,179, with country-specific sample sizes ranging from 711 to 1801. Some studies separated outcomes based on specific orphan subtypes, including maternal, paternal and double orphanhood; we describe these subgroup comparisons in the text where appropriate.

### Methodological appraisal of included studies

Methodological quality among included studies was generally strong. Nine of 10 studies used representative or systematic sampling techniques to recruit participants [29,30,32-38]. Only one study used convenience sampling [31]. Studies were inconsistent, however, in their targeted sample; three included only females [29,31,35], and sample age ranges varied. All studies provided an explicit definition for orphan status, generally adhering to the Joint United Nations Programme on HIV/AIDS (UNAIDS) definition as death of one or more parent. Some studies provided subgroup classifications and comparisons for maternal, paternal and double orphans [29-31,33,35,37]; however, this was not consistent.

HIV infection was determined through biological test data in six studies [29-32,34,38]; two studies also tested for HSV-2 infection [29,31] and one study conducted pregnancy testing [31]. All but one study [38] assessed self-reported sexual risk behaviour, with notable differences in measures and recall periods between studies. This variability prevented a meta-analysis of self-reported behaviour data. Sexual behaviour data from one study could not be disaggregated by orphan status, so they are not reported here [32]. Six studies reported participation rates [29-32,34,36], which ranged from 67% to 98%. All but one study [38] used multivariate analyses to test associations between orphanhood and HIV or sexual risk behaviours, controlling for relevant socio-demographic co-factors. Studies were inconsistent in whether they analyzed data for males or females separately or analyzed the entire sample with gender as a covariate; analytic approach varied according to the intended aim of the paper.

### Meta-analysis of HIV prevalence in orphaned versus non-orphaned participants

**Figure 2:**
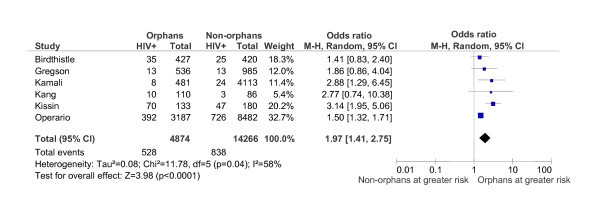
**Meta-analysis of six studies (n = 19,140) comparing HIV-positive serostatus in orphaned versus non-orphaned youth**.

Six studies conducted HIV-testing in a total of 19,140 participants (4874 classified as orphaned and 14,266 as non-orphaned) [29-32,34]. Crude non-weighted HIV prevalence was 10.8% (n = 528) in participants who reported any parental death and 5.9% (n = 838) in participants who reported both parents alive. Figure 2 shows weighted ORs and 95% CIs for HIV prevalence in each study.

Results from a random-effects meta-analysis indicated significantly greater HIV prevalence in orphaned participants compared with non-orphans (OR = 1.97; 95% CI = 1.41-2.75). Between-study heterogeneity was not significant, indicated by a χ^2 ^value of 7.97 (p = 0.16) and I^2 ^value of 37%. Using Oswald's fail-safe N formula, 27 null-effect studies would be needed to invalidate the significant meta-analytic effect. The funnel plot of effect sizes was somewhat asymmetrical, suggesting the absence of smaller studies with an OR less than 1.97 or studies with a less precise estimate of association between orphanhood and HIV.

### Incidence of STIs other than HIV

Four studies (n = 13,478) evaluated the incidence of sexually transmitted infections (STIs) other than HIV, including HSV-2 [29,31], self-reported history of STI symptoms [30], and self-reported STI in the past year [34]. One study (n = 653) found significantly greater prevalence of HSV-2 infection among both maternal and paternal orphan subgroups, but not for double orphans compared with non-orphans [29]. The remaining studies found no significant differences for maternal orphans [30,31], paternal orphans [30,31] or all orphan subtypes [31,34] compared with non-orphans.

### Pregnancy

Study findings for pregnancy and STI outcomes are presented in Table 2. Five studies (n = 22,398), including the 10-country study, assessed whether female participants had ever been pregnant [29,30,34,35] or tested female participants for pregnancy during the study [31]. These studies found significantly greater risk for pregnancy among maternal orphans [29-31,35], paternal orphans [35], double orphans [35] and all orphan subtypes combined [34,35] compared with non-orphans. Results in the 10-country study reached significance among all orphans in Chad and Cote d'Ivorie, maternal orphans in Cote d'Ivoire, paternal orphans in Chad, and double orphans in Benin [35].

**Table 2:** STI and pregnancy among orphans versus non-orphans

**Study**	**n **♂♀	**All orphans vs. non-orphans**		**Maternal orphans vs. non-orphans**		**Paternal orphans vs. non-orphans**		**Double orphans vs. non-orphans**	
		**STI other than HIV**	**Pregnancy**	**STI other than HIV**	**Pregnancy**	**STI other than HIV**	**Pregnancy**	**STI other than HIV**	**Pregnancy**
					
Birdthistle [29]	863 ♀			aOR = 5.9 (2.2-15.7)	aOR = 3.7 (1.0-14.0)	aOR = 3.5 (1.5-8.4)	ns	ns	ns
Gregson [30]	1523 ♂♀			ns	aOR = 1.98 (1.05-3.74)	ns	ns		
Kang [31]	196 ♀	ns	ns	ns	aOR = 3.14 (1.17-8.43)	ns	ns		
Operario [34]	11,904♂♀	ns	aOR = 1.15 (1.01-1.34)						
Palermo [35] Benin	1801 ♀		ns		ns		ns		aOR = 2.62
Palermo [35] Chad	884 ♀		aOR = 1.69		ns		aOR = 1.83		ns
Palermo [35] Congo	914 ♀		ns		ns		ns		ns
Palermo [35] Cote d'Ivoire	711 ♀		aOR = 1.69		aOR = 2.57		ns		ns
Palermo [35] Lesotho	1043 ♀		ns		ns		ns		ns
Palermo [35] Malawi	1337 ♀		ns		ns		ns		ns
Palermo [35] Mozambique	1484 ♀		ns		ns		ns		ns
Palermo [35] Tanzania	1375 ♀		ns		ns		ns		ns
Palermo [35] Uganda	1219 ♀		ns		ns		ns		ns
Palermo [35] Zimbabwe	1207 ♀		ns		ns		ns		ns

aOR = adjusted odds ratio with 95% confidence interval. ns = non-significant result. Odds ratios >1 indicate that orphans had significantly higher odds of STI or pregnancy. Confidence intervals were not available for the study by Palermo *et al *[35]. This table uses adjusted odds ratios rather than risk ratios because odds ratios were reported consistently throughout the primary studies, and we had insufficient data to transform them; we report adjusted odds ratios here as calculated in the primary studies.

### Sexual behaviours

Sexual behaviour findings are organized by orphan subtype, reflecting how they were reported in the primary studies: all types of orphans combined, maternal orphans, paternal orphans and double orphans. These findings are summarized in Table 3, along with the number and gender of participants for each study.

**Table 3:** Sexual risk behaviours of orphans versus non-orphans, by orphan subgroup

**Study**	**n **♂♀	**All orphans vs. non-orphans**					**Maternal orphans vs. non-orphans**					**Paternal orphans vs. non-orphans**					**Double orphans vs. non-orphans**				
		**US**	**S**	**MP**	**FS**	**TS**	**US**	**S**	**MP**	**FS**	**TS**	**US**	**S**	**MP**	**FS**	**TS**	**US**	**S**	**MP**	**FS**	**TS**
					
Birdthistle [29]	863 ♀						ns	*	*	ns	ns	**†**	ns	ns	ns	ns	**†**	ns	*	ns	ns
Gregson [30]	1523 ♂♀							*	ns				ns	ns							
Kang [31]	196 ♀	ns	*	ns	ns	ns	ns	ns	ns	**†**	*	ns	ns	ns	ns	ns					
McGrath [37]	8753 ♂♀							*					*								
Nyamukapa [33]	4660 ♂♀						ns	*		ns		*♂	*		*		ns	ns		ns	
Operario [34]	11,904 ♂♀	*♂	*	*♀	ns	ns															
Palermo [35] Benin	1801 ♀		ns					ns					ns					*			
Palermo [35] Chad	884 ♀		ns					ns					ns					ns			
Palermo [35] Congo	914 ♀		ns					ns					ns					ns			
Palermo [35] Cote d'Ivoire	711 ♀		*					ns					*					ns			
Palermo [35] Lesotho	1043 ♀		*					ns					*					*			
Palermo [35] Malawi	1337 ♀		ns					ns					ns					*			
Palermo [35] Mozambique	1484 ♀		*					ns					*					ns			
Palermo [35] Tanzania	1375 ♀		*					*					ns					ns			
Palermo [35] Uganda	1219 ♀		ns					*					ns					ns			
Palermo [35] Zimbabwe	1207 ♀		ns					ns					ns					ns			
Thurman [36]	1694 ♂♀	ns	*	ns	*	*															

n = total number of participants. US = unprotected sex. S = ever had sex. MP = multiple partners in lifetime. FS = forced or unwilling sex. TS = transactional sex. * = significant difference with orphans reporting higher risk than non-orphans. ns = no significant difference between orphans and non-orphans. *♀ = significant among females but not males, orphans reporting higher risk. *♂ = significant among males but not females, orphans reporting higher risk. † = significant difference with non-orphans reporting higher risk than orphans.

#### All orphans

Four assessed unprotected sex, defined as condom or contraceptive non-use at first sex [31] or last sex [34-36]; one found significantly greater risk among male orphans compared with male non-orphans [34]. Four studies assessed sexual debut [31,34-36], two of which defined sex as oral, anal or vaginal [31,34]; all four found that orphans were significantly more likely to have experienced sexual debut than non-orphans. Findings were significant in four sites of the 10-country survey (Cote d'Ivoire, Lesotho, Mozambique and Tanzania) [35].

Three studies assessed participant reports of multiple sexual partners, with recall periods of the participants' lifetime [31] or past year [34,36]; one found that female orphans were more likely to have multiple partners than female non-orphans [34], while other findings were non-significant. The same three studies assessed forced or unwilling sex ever [34] or at first sex [31,36]; one of these found a significantly greater likelihood of forced or unwilling sex among orphans compared with non-orphans [36]. The same three studies assessed transactional sex, defined as ever exchanging sex [34,36] or receiving basic needs from a current sexual partner [31]; results in one study indicated significantly greater risk among orphans than among non-orphans [36].

#### Maternal orphans

Six evaluations reported sexual behaviours for maternal orphans [29-31,33,35,37]. Three assessed unprotected sex, defined as unprotected first sex [29-31] or high-risk sex [33], and none found a significant difference between orphans and non-orphans. All six assessed sexual debut; one assessed age of first sex [37] and another defined sex as oral, anal or vaginal [31]. Five of the six found significant differences indicating a higher risk among orphans [29,30,33,35,37]. Findings were significant at two sites of the 10-country study (Tanzania and Uganda) [35].

Three studies assessed whether participants reported multiple lifetime sexual partners [29-31]; one found significantly greater risk among orphans [29]. Three studies assessed forced sex ever [29,33] or at first sex [31]; unexpectedly, the one significant finding was that maternal orphans were less likely to experience forced sex than non-orphans [31]. Two studies reported either transactional sex [29] or receipt of basic needs from a current sexual partner [31]; one of these found that maternal orphans were at significantly greater risk than non-orphans.

#### Paternal orphans

Six evaluations reported sexual behaviours for paternal orphans [29-31,33,35,37]. Three assessed unprotected sex using measures already described [29,31,33]; one found a significantly protective association between paternal orphanhood and unprotected sex [29], while another found significantly greater risk among male paternal orphans than among male non-orphans [33]. All six studies assessed sexual debut, using measures that we have described; three found that paternal orphans were significantly more likely to have had sex than non-orphans [33,35,37].

Findings were significant in three sites of the 10-country survey (Cote d'Ivoire, Lesotho and Mozambique) [35]. Three studies assessed whether participants reported multiple lifetime sexual partners, none of which found a significant effect [29-31]. Three assessed forced sex [29,31,33]; one of these found that paternal orphans were significantly more likely to have experienced forced sex than non-orphans [33]. Two assessed transactional sex as defined [29,31]; neither found a significant difference between paternal orphans and non-orphans.

#### Double orphans

Three studies reported sexual behaviours for double orphans [29,33,35]. Two assessed unprotected first sex [29] or high-risk sex [33]; one found a protective association between double orphanhood and unprotected sex [29]. All three assessed sexual debut, and the 10-country study found significantly greater risk among double orphans than non-orphans; this finding reached significance in Benin, Lesotho and Malawi [35]. The one study to measure multiple lifetime sexual partners found that double orphans were significantly more likely to have had multiple partners than non-orphans [29]. Two studies assessed forced sex ever [29,33]; neither found a significant difference between double orphans and non-orphans. Similarly, the only study to measure transactional sex found no significant association between double orphanhood and risk [29].

## Discussion

This analysis aimed to examine whether orphaned youth experience greater risk for HIV infection compared with their non-orphaned peers. Our research covered 10 studies representing participants in 12 countries, mostly in sub-Saharan Africa, which included 46,856 participants and conducted HIV testing in 19,140 participants. Based on a meta-analysis of identified studies, we estimated that orphaned youth experience nearly two-fold greater odds for testing positive for HIV, which provided support for our hypothesis that orphans are at greater risk of HIV infection.

Although studies varied in the measurement and reporting of STIs, pregnancy and sexual risk behaviours among orphans versus non-orphans, the direction of significant effects generally showed greater sexual risk among orphaned youth compared with non-orphans. Due to inconsistencies among studies in measurement items, reporting and time frames, meta-analysis of self-reported risk behaviours was not possible.

Strengths of this research include its international scope, systematic search strategy, appraisal of methodological quality and meta-analysis of HIV prevalence. All but one study reported data from sub-Saharan Africa, the region that carries the heaviest burden of HIV and AIDS-related deaths globally. One identified study, conducted in St Petersburg, Russia, represents a different epidemiological profile; HIV/AIDS in Russia is more likely to be associated with injection drug use compared with sub-Saharan Africa, where heterosexual transmission accounts for the majority of infections. Notably, no relevant studies were identified from Asia, where there is a rapidly growing HIV epidemic and an escalating orphanhood problem [3].

Trends across studies suggest that female orphaned youth might be particularly at risk for HIV infection. Of the six studies included in our comparative meta-analysis of HIV prevalence between orphans and non-orphans, two studies included only females [29,31] and two studies including both males and females found greater HIV seroprevalence only among female orphans [30,34]. Results from three national representative studies (in Chad, Cote d'Ivoire and South Africa) showed that female orphans were significantly more likely to have been pregnant than female non-orphans.

Potential factors that increase female orphan youth's vulnerability for HIV and related health and social problems have been described elsewhere [2,3,5,10,11]. Due to the loss of adults in the household, female orphaned youth might experience pressure to generate household income or assume adult responsibilities, such as family caregiving. Female orphaned youth might also be at greater risk for educational shortfalls, such as discontinuation and poor performance, due to competing household responsibilities. In turn, these factors - school drop-out, early adult responsibilities, economic pressure - might be associated with sexual risk behaviour for female orphan youth.

It is difficult to make comparisons in HIV risk by orphan subtype (e.g., maternal versus paternal versus double orphans). Meta-analysis of HIV seroprevalence was not conducted by orphan subtype because studies did not consistently compare different types of orphanhood status. However, one trend emerged in Table 2, suggesting that maternal female orphans appeared more likely to have been pregnant, based on results from four studies; this finding was not as strong for paternal orphans. Table 3 shows further that maternal orphans were more likely to have experienced sexual debut, based on results from five studies (conducted in South Africa, Tanzania, Uganda and Zimbabwe), and more likely to report multiple partners and transactional sex in studies conducted in Zimbabwe.

However, paternal orphans were also more likely to have experienced sexual debut in three studies (conducted in Cote d'Ivoire, Lesotho, Mozambique, South Africa and Zimbabwe), and more likely to report unprotected sex (females only) and forced sex in one study conducted in Zimbabwe, as is evident in Table 3. Fewer studies reported findings for double orphans. Future studies should more consistently report comparisons by orphan subtype in order to determine whether type of orphanhood is associated with level of risk.

The fact that many findings did not reach statistical significance highlights complexity in the measurement of orphan status and in measures of sexual risk behaviour and STI outcomes, which has been observed in other studies [40]. Studies might have been challenged in validating parental death and determining cause of parental death [6]. Some studies may be limited by floor effects (e.g., forced sex) or ceiling effects (e.g., sexual debut, especially when participants are generally older teens). Additionally, the measures used for sexual behaviour throughout these studies may not have isolated the behaviours most indicative of HIV risk. Very few of the included studies reported on specific types of sex act (vaginal, anal or oral sex) or types of partners (e.g., sex workers, partners with a large age difference). Measurements were often limited to participants' first or most recent sexual encounter (e.g., forced sex at first sex, condom non-use at first sex).

Studies generally did not report on participants' partner characteristics (e.g., having older partners, riskier partners), which might be more likely to determine actual risk for HIV transmission than sexual behaviours *per se*. Future studies should consider using more precise definitions for behavioural measurements for sexual risk and more consistent measurement and reporting of orphan status and orphan group subtype.

Studies identified for this review showed many methodological strengths and some noteworthy limitations. Nine of 10 studies used representative or systematic sampling to minimize recruitment bias; reported participation rates were moderate to high. Biological specimens were collected in six studies. All studies but one used multivariate analysis to assess the independent association of orphan status on HIV risk, controlling for potential confounding variables.

However, studies were inconsistent in comparing subgroups, such as maternal, paternal and double orphanhood. Samples also varied between studies in gender composition; three studies included only females. Only one study provided a theoretical framework to explain the association between orphanhood and HIV risk [33]. No studies reported on age at which participants experienced parental death, which can be an important developmental co-factor for risk behaviours in orphaned youth.

Limitations to the conclusions drawn from this systematic review must be considered. First, cause of HIV infection in the seroprevalence studies could not be determined directly, although likelihood of perinatal infection is low for the age groups tested. Second, no studies assessed cause of parental death, thereby limiting any conclusion about effects of parental AIDS death on children. Third, due to the wide age range included in this analysis, we are unable to specify developmental timing for specific risks. Timing of parental death (e.g., during early adolescence) and length of orphanhood might be important determinants of HIV risk, but were not reported in identified studies.

Fourth, data included in this review were cross-sectional, and therefore we cannot infer temporal or causal relationships between parental death, HIV infection and self-reported risk behaviour. Indeed, although the meta-analysis indicated that orphans show greater HIV seroprevalence and qualitative synthesis suggested that orphans might engage in riskier behaviours, we cannot determine why these associations might exist. For example, partner characteristics, such as having older partners, might confer a substantial amount of risk to orphaned children.

Fifth, despite our comprehensive and systematic literature search, this review might not have identified all relevant studies. Sixth, because most identified studies were conducted in sub-Saharan Africa, findings might not be generalizable to other geographic areas. Seventh, one study accounted for the majority of participants included in this meta-analysis [34]. However, after omitting this study from the meta-analysis, the overall effect remained significant (RR = 1.88; 95% CI = 1.49-2.36) suggesting the association between orphan status and HIV serostatus is robust.

## Conclusions

Evidence from this review suggests a need for HIV prevention interventions to address orphaned youth, particularly in sub-Saharan Africa, where most studies were conducted.

Findings provide further empirical support to previous reports and narrative reviews on the greater risk for HIV infection through sexual transmission in orphaned children and young people [2,3,23]. Although this study could not directly determine the mechanisms linking orphanhood and sexual risk behaviour, which might be targeted in prevention and counselling efforts, literature suggests possible co-factors, such as increased educational shortfalls [9,23], psychological problems [17], economic difficulties [20] and family disruptions [41]. The sequelae of parental death are likely to differ according to gender, age of child at orphanhood, presence of other surviving family members, and geographic region [5,6].

To design effective public health responses for these youth, we need a clearer understanding of the consequences of parental death in the context of AIDS. Moreover, a stronger understanding of protective factors is necessary to develop public health interventions that build on the strengths of youth, families and communities affected by AIDS.

## Competing interests

The authors declare that they have no competing interests.

## Authors' contributions

All authors contributed to the manuscript and approved the final version. DO conceptualized the review and led the writing. KU and CC were involved in conducting the literature search, data extraction, data synthesis and analysis. LC was involved in interpreting the findings.
